# A Zero‐Energy, Zero‐Emission Air Conditioning Fabric

**DOI:** 10.1002/advs.202206925

**Published:** 2023-02-15

**Authors:** Kai Zhang, Xiaojuan Lei, Caiqing Mo, Jin Huang, Ming Wang, En‐Tang Kang, Liqun Xu

**Affiliations:** ^1^ School of Materials and Energy Chongqing Key Laboratory of Soft‐Matter Material Chemistry and Function Manufacturing Southwest University Chongqing 400715 P. R. China; ^2^ College of Food Science Chongqing Key Laboratory of Soft‐Matter Material Chemistry and Function Manufacturing Southwest University Chongqing 400715 P. R. China; ^3^ School of Chemistry and Chemical Engineering Chongqing Key Laboratory of Soft‐Matter Material Chemistry and Function Manufacturing Southwest University Chongqing 400715 P. R. China

**Keywords:** absorption/evaporation, asymmetric bilayer structure, fabric, moisture/thermal management, power generator

## Abstract

High indoor humidity/temperature pose serious public health threat and hinder industrial productivity, thus adversely impairing the wellness and economy of the entire society. Traditional air conditioning systems for dehumidification and cooling involve significant energy consumption and have accelerated the greenhouse effect. Here, this work demonstrates an asymmetric bilayer cellulose‐based fabric that enables solar‐driven continuous indoor dehumidification, transpiration‐driven power generation, and passive radiative cooling using the same textile without any energy input. The multimode fabric (ABMTF) consists of a cellulose moisture absorption–evaporation layer (ADF) and a cellulose acetate (CA) radiation layer. The ABMTF exhibits a high moisture absorption capacity and water evaporation rate, which quickly reduces the indoor relative humidity (RH) to a comfortable level (40–60% RH) under 1 sun illumination. The evaporation‐driven continuous capillary flow generates a maximum open‐circuit voltage (*V*_oc_) of 0.82 V, and a power density (*P*) up to 1.13 µW cm^−3^. When a CA layer with high solar reflection and mid‐infrared (mid‐IR) emissivity faces outward, it realizes subambient cooling of ≈12 °C with average cooling power of ≈106 W m^−2^ at midday under radiation of 900 W m^−2^. This work brings a new perspective to develop the next‐generation, high performance environmentally friendly materials for sustainable moisture/thermal management and self‐powered applications.

## Graphical abstract

A self‐powered moisture/thermal management fabric is constructed by integrating moisture absorption/evaporation layer and radiative cooling layer. In high humidity environments, the fabric can unidirectionally exhaust the indoor moisture to a comfortable level under solar illumination, and achieve synergistic power generation. At high temperature, the spectrally selective radiation layer maximizes heat loss and enables the fabric to cool below the ambient temperature.

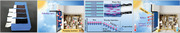

## 1 Introduction

Buildings account for approximately 40% of global energy consumption and almost one‐third of global CO_2_ emissions, being a critical piece of our transition to a lower‐carbon future.^[^ ^1^, ^2^ ^]^ Reports from the United Nations have pointed out that nearly 70% of the world will live in urban areas by 2050.^[^ ^3^ ^]^ Increasing building densities and indoor activity levels highlight the importance of indoor moisture/thermal regulation. Traditional air conditioning systems remove moisture by cooling air below its dew point and then reheating it to the desired temperature, while relieve heat stress by cooling the air. Evidence suggests that more than half of building energy consumption, equivalent to nearly 20% of total energy consumption, comes from air conditioning systems.^[^ ^4^, ^5^, ^6^ ^]^ This specially makes the growing energy crisis and greenhouse effect continue to worsen.^[^ ^7^ ^]^

Solar radiation, as well as convection and conduction of hot outdoor air, are the main heat loads that cause the indoor air in buildings to warm up, and mid‐infrared (mid‐IR) radiation is the main energy flow that counteracts this trend.^[^ ^8^, ^9^ ^]^ Similarly, the outdoor moisture input and hot air condensation are the main factors in the generation of high indoor humidity.^[^ ^10^, ^11^ ^]^ The unbalanced energy flows and moisture transport result in high indoor temperature/humidity. Therefore, it is highly desirable to explore a sustainable zero‐energy and zero‐emission air conditioning technology to balance the energy flow and moisture transport of indoor air. On the one hand, radiative cooling technology that exploit mid‐IR emission at the atmospheric transparent window to directly transfer heat from ambient objects to the cold universe (3 K) represents a promising strategy for balancing indoor thermal flows.^[^ ^12^, ^13^, ^14^ ^]^ On the other hand, it is worth noting that moisture is usually transported autonomously from high to low humidity surroundings. However, under the driving force of thermal gradients, moisture can also be transported in the opposite direction from the low humidity case to high humidity case.^[^ ^15^, ^16^, ^17^ ^]^ In summer's high radiation weather, solar energy is a rich source of energy that cannot be ignored.^[^ ^18^, ^19^ ^]^ Solar‐driven steam generation have inspired more structure/function designs and an interdisciplinary effort for advanced energy‐harvesting materials and devices. Thus, it is particularly important and imperative to develop an environmental moisture/thermal management material through manipulating energy flow and moisture transport, which can use solar energy to drive reverse indoor moisture removal via photothermal evaporation, and achieve passive cooling via mid‐IR radiation and solar reflectance.

Fabrics are developed to cover a variety of application needs, including flexible conductors,^[^ ^20^, ^21^ ^]^ sweat wicking,^[^ ^22^ ^]^ water collecting,^[^ ^23^, ^24^ ^]^ and water transportation^[^ ^25^, ^26^ ^]^ due to their easy‐availability, hydrophilicity, porosity, and breathability. Zhang et al. developed an atmosphere water harvesting fabric by using cellulose fabric's multiscale cellulose network and adsorption–condensation mechanism.^[^ ^27^ ^]^ The obtained fabric exhibits 8.3% solar absorption and ≈0.9 IR emissivity that enable cooling up to 7.5 °C below ambient temperature with energy‐free radiative cooling. Li et al. successfully fabricated a hierarchically structured cellulose acetate (CA) radiative cooling fabric by using electrostatic spinning technique, which can inhibit the melting of ice under sunlight.^[^ ^28^ ^]^ Inspired by the transpiration of natural trees, Zhang et al. developed a double‐layer electrospinning fabric consisting of a desiccant layer and a photothermal layer, which can be used as an energy‐efficient solar‐powered moisture pump to effectively reduce indoor humidity to a comfortable level of 45%‐65%.^[^ ^16^ ^]^ Miao et al. designed a Janus wettable bilayer polyurethane/silicon nitride fabric that integrates multimode cooling and unidirectional perspiration functions.^[^ ^29^ ^]^ While fabrics are increasingly used for passive cooling, water transport, and collection, no indoor moisture/thermal regulation technologies or fabrics based on a combination of radiative cooling and solar‐driven dehumidification have been reported.

Herein, we report an asymmetric bilayer design of indoor air conditioning fabric using a facile and scalable two‐step dip‐coating and spraying method for adaptive indoor moisture/thermal management. The developed air conditioning fabric consists of moisture adsorption‐evaporation layer (cellulose/Zn‐complex/a‐MWCNTs) and radiative CA layer. To build a continuously operating solar‐driven moisture pump, a hydrophilic and easily accessible commercial cellulose fabric was chosen as the moisture transport substrate. The highly hygroscopic Zn‐complex nanosheets and carboxylated multiwalled carbon nanotubes (a‐MWCNTS) were impregnated on both sides of the length direction as the indoor moisture adsorption regions and outdoor photothermal evaporation regions, respectively. In high‐humidity environments, the desiccant zone with high specific surface area and porosity displays an excellent moisture absorption capacity of 2.05 g g^−1^ at 25 °C and 90% RH, quick moisture absorption and transport rates, allowing indoor moisture to be collected. The evaporation zone exhibits a high solar absorption of 97%, efficient solar thermal conversion, and good moisture permeability, thus promoting water evaporation and ensuring stable and continuous moisture removal. During this process, local moisture absorption and evaporation on both sides of the moisture pump encourage the formation of a naturally gradient wetting carbon structure in the photothermal zone, achieving synergistic power generation. At high temperature, the reverse sprayed CA radiant layer exhibits a high mid‐IR emissivity because of its inherent molecular bonding vibrations, and scatters sunlight due to the designed porous structure. As a result, the protected building's heat load is effectively reduced, and a passive cooling effect is achieved. This robust asymmetric bilayer cellulose‐based fabric with performable dehumidification (power generation)/cooling switch not only provides an attempt for real‐time indoor comfort regulation, but also brings a new perspective on comprehensive solar utilization.

## 2 Results and Discussion

### 2.1 Transpiration Combined with Radiation Cooling‐Inspired Moisture/Thermal Management Fabric

Plants use transpiration to pump water absorbed by the roots from the soil up to the vegetative stem, where it is further evaporated by the branches and leaves.^[^ ^30^, ^31^ ^]^ Inspired by this naturally unique and delicate characteristics, a biomimetic sunlight‐driven asymmetric dehumidification fabric has been developed for indoor moisture absorption and evaporation. Cellulose, which exists as the main structural component of plant cell walls, is the most abundant hydrophilic polymer on earth, featuring strong water absorption, low cost, and low weight. Aside from being a potentially sustainable material, cellulose enables a variety of functions and transformative applications due to its unique multidimensional structure and integration with functional materials. Notably, cellulose has been regarded as attractive candidates in the fields of mass and heat transfer, including water transport (fluid materials), interfacial photothermal evaporation, ion transport, thermal energy harvesting, and radiation cooling.^[^ ^32^, ^33^ ^]^

**Figure 1:**
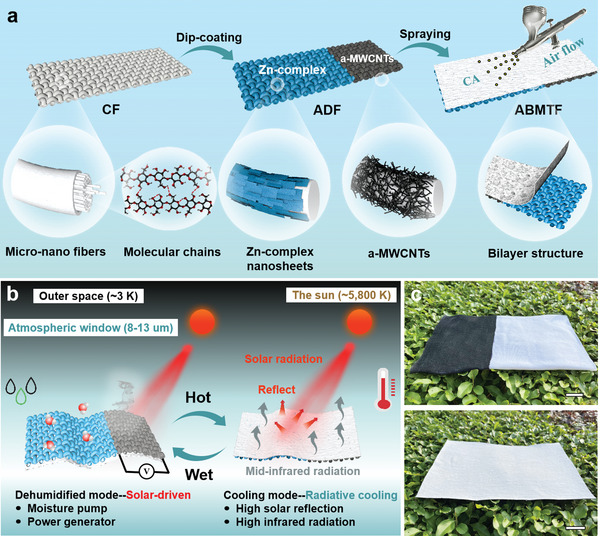
Fabrication and working mechanism of the multimode moisture/thermal management fabric. a) Schematic illustration of the fabrication of asymmetric bilayer ABMTF. b) Schematic of indoor moisture adsorption/evaporation, power generation and radiative cooling mechanism of ABMTF. c) Optical images of ABMTF in dehumidification/power generation and cooling modes. The scale bar is 3 cm.

Cellulose fabric possesses an orderly woven porous microfiber network structure with excellent water wettability, and the large number of naturally occurring micro‐ and nanostructured fibers are very conducive to water transport.^[^ ^27^, ^34^ ^]^ As shown in **Figure** **1**a, the dehumidification fabric was prepared firstly by partially impregnating the cellulose fabric with a‐MWCNTs, and the impregnated cellulose/a‐MWCNTs fabric effectively introduced a light‐absorbing coating, showing an obvious asymmetric structure. Secondly, the unimpregnated side of cellulose/a‐MWCNTs fabric was poured with Zn‐complex nanosheet dispersion to obtain asymmetric cellulose/Zn‐complex/a‐MWCNTs dehumidified fabric (ADF). Highly hygroscopic Zn‐complex nanosheets were synthesized using a time‐saving and cost‐effective coordination reaction between ethanolamine ligands and zinc chloride salts. Due to the highly porous structure and hydroxyl groups of cellulose fabric, Zn‐complex nanosheets can effectively penetrate the fiber backbone and adhere tightly to the surface layer through ionic and hydrogen bonds. Subsequently, the porous CA radiation layer was sprayed on one side of ADF to construct an asymmetric bilayer cellulose/Zn‐complex/a‐MWCNTs/CA moisture/thermal management fabric (ABMTF).

The structural schematic diagram, moisture transport channels and heat radiation paths of the ABMTF with integrated moisture absorption–evaporation, power generation and radiation cooling functions are shown in Figure 1b. The ABMTF has interconnected water transport channels for moisture absorption and photothermal vapor conversion. In high‐humidity environments, the desiccant‐loaded zone (cellulose/Zn‐complex) can adsorb moisture from air, and then the captured moisture is desorbed into water vapor through irradiating the photothermal zone (cellulose/a‐MWCNTs). Therefore, using a solar‐driven moisture transport fabric enables efficient and consistent indoor dehumidification. The formation of a naturally gradient wetting carbon structure during this process results in synergistic power generation. At high temperature, the sprayed CA layer exhibits a high sunlight reflectance due to its highly porous scattering structure, and a high emissivity in the mid‐IR region owing to its specific molecular vibrations. This spectrally selective radiation layer maximizes heat loss and enables the fabric to achieve a cooling effect below the ambient temperature. Figure 1c shows the images of ABMTF with a‐MWCNTs and Zn‐complex nanosheets impregnated onto cellulose fabric. The left evaporation zone shows a deep black surface, which is favorable for high vapor conversion efficiency. The right moisture absorption zone requires a high moisture absorption rate, and the Zn‐complex nanosheets meet this criterion by allowing moisture to quickly infiltrate the cellulose fabric after absorbing moisture. The other side of ABMTF sprayed with CA radiant layer appears bright white, which promotes the reflection of sunlight. Benefited from this, the fabric shows a substantial difference in visual appearance between dehumidification and cooling modes.

### 2.2 Morphological and Structural Characterization of Asymmetric Moisture/Thermal Management Fabric

**Figure 2:**
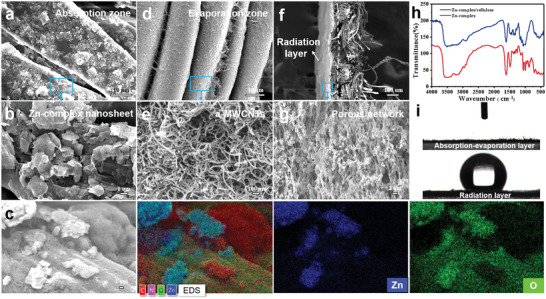
Morphology and structure characterization of the moisture/thermal management fabric. a,b) FE‐SEM images of moisture absorption zone (Zn‐complex/cellulose). c) FE‐SEM image and corresponding elemental maps of moisture absorption zone. d,e) FE‐SEM images of evaporation zone (a‐MWCNTs/cellulose). f,g) FE‐SEM images of porous cellulose acetate (CA) radiation layer (surface). h) FTIR spectra of the absorption zone and Zn‐complex. i) The water contact angle (WCA) of absorption–evaporation layer and radiation layer.

Cellulose fabrics are very easy to prepare on a large scale and are widely used in everyday life. The microstructures of the asymmetric moisture/thermal management fabric are shown in **Figure** **2**. The cellulose fabric has a multiscale structure consisting of an interconnected and ordered microfiber network. The tight physical entanglement of fibers provides ideal flexibility and mechanical strength for macroscopic fabrics (Figure S1, Supporting Information). Micron‐scale cellulose filaments are further composed of ordered cellulose nanofibers.^[^ ^27^ ^]^ This well‐ordered porous structure resembles the morphology of wood in the vertical growth direction and provides a good substrate for water transport and evaporation.^[^ ^33^, ^34^, ^35^, ^36^ ^]^After impregnation of one end with Zn‐complex dispersion, the Zn‐complex nanosheets were uniformly and stably attached to the surface of cellulose microfibers, indicating that the continuous moisture absorption zone was successfully constructed on the fabrics (Figure 2a,b). The effective distribution of Zn, O, and C elements in Zn‐complex nanosheets on cellulose microfibril surface was observed by field emission scanning electron microscopy (FE‐SEM) and corresponding elemental imaging maps, demonstrating that Zn‐complex nanosheets were successfully loaded on the fiber surface (Figure 2c). The other side of the fabric was impregnated with a‐MWCNTs dispersion to introduce photothermal evaporation zone. FE‐SEM images show that a‐MWCNTs effectively coat on the microfiber surface. This fiber network's large light‐absorbing area facilitates sunlight absorption and vapor generation without disrupting the penetrating network structure (Figure 2d‐e).

The FTIR spectra of Zn‐complex and moisture absorption zone exhibit a series of absorption peaks in the range of 400–4000 cm^−1^, and a broad band from 3100 to 3600 cm^−1^ was assigned to OH stretching bonds of cellulose (Figure 2h). The other cellulose characteristic peaks are at 1450 cm^−1^ (C−H, O−H bending), 1332 cm^−1^ (O−H and C−H oscillation), and 1098 cm^−1^ (C−O stretching), respectively.^[^ ^37^, ^38^ ^]^ The FTIR spectrum of Zn‐complex shows a characteristic peak of Zn−O bond at 512 cm^−1^, which is also present in ADF's absorption zone.^[^ ^39^, ^40^ ^]^ In addition, the amino and hydroxyl peaks of the complex' ethanolamine portion are present in the spectral curve, with amino absorption peaks at 3501 and 1616 cm^−1^, and hydroxyl absorption peaks at 3237 cm^−1^.^[^ ^41^, ^42^ ^]^ The FTIR spectrum of moisture absorption zone contains typical cellulose and Zn‐complex characteristic peaks, indicating that Zn‐complex nanosheets are well assembled on the cellulose fabric. On the other hand, as a radiant layer, we sprayed a 85 µm thick CA coating on one surface of the ADF. Figure 2f shows that the formed ABMTF exhibits an obvious bilayer structure, with the sprayed layer consisting of penetrating nanopores (Figure 2g and Figure S2, Supporting Information), so the air and vapor permeability of the fabric can be maintained. The WCA of evaporation side and radiant layer of ABMTF are 0° and 130°, respectively, verifying its amphiphilic Janus characteristics (Figure 2i).

### 2.3 Moisture Absorption and Photothermal Evaporation Properties of Moisture Management Fabric

**Figure 3:**
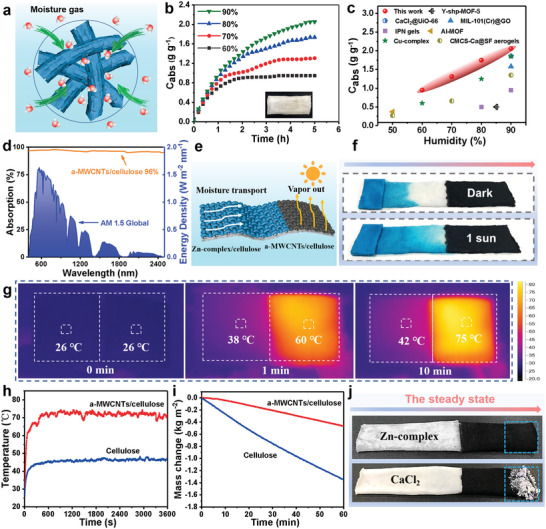
Moisture adsorption and photothermal behaviors of the moisture management fabric. a) Schematic showing the moisture adsorption behaviors of cellulose/Zn‐complex (adsorption zone). b) Moisture adsorption kinetics of adsorption zone at 23 °C and various relative humidity (RH). c) A comparison chart showing the moisture adsorption capability of some reported representative desiccant materials: Y‐shp‐MOF‐5,^[^ ^43^ ^]^ CaCl_2_@UiO‐66,^[^ ^44^ ^]^ MIL‐101(Cr)@GO,^[^ ^45^ ^]^ IPN gels,^[^ ^46^ ^]^ Al‐MOF,^[^ ^47^ ^]^ Cu‐complex,^[^ ^41^ ^]^ and CMCS‐Ca@SF aerogels.^[^ ^48^ ^]^ d) Ultraviolet–visible–near infrared (UV‐Vis‐NIR) absorption spectra for the photothermal zone and the solar spectrum (AM 1.5G). e) Schematic displaying the moisture transport and vapor‐out mechanisms of ADF. f) Water diffusion control by illuminating the evaporation zone of ADF. It was demonstrated that the introduction of sunlight irradiation extend the water diffusion distance due to accelerated water flow. Scale bar:1 cm. g) IR thermal images of the surface temperature change of cellulose/a‐MWCNTs (photothermal zone) and pure cellulose textile under light intensity of 1 kW m^−2^. h) Detailed surface temperature evolution of fabrics under one sun illumination. i) Time‐dependent water evaporation from the fabric under one sun illumination. j) Salt accumulation behavior of moisture management fabrics loaded with different desiccants after 5 h of continuous evaporation under 1 sun radiation.

**Figure** **3**a shows the behavior of ADF in absorbing moisture and infiltrating liquid water. Water vapor molecules are captured and liquefied at the Zn‐complex nanosheet surface. Subsequently, water diffuses into the porous cellulose network, allowing water to be trapped from the moist air. The hydrophilic micro‐nano‐fiber backbone facilitates water storage. As a result, the ADF is able to capture liquid water directly from moist air by absorbing water vapor without consuming energy (no latent heat of evaporation and condensation). Figure 3b illustrates the ADF's adsorption curves at 25 °C and various relative humidity (RH). The measured water absorption capacities reach 0.96, 1.31, 1.73, and 2.05 g g^−1^ at 60%, 70%, 80%, and 90% RH, respectively. The fabric is able to rapidly absorb moisture from humid air in 1h. As the RH increases, the time required to completely saturate the fabric rises significantly, mainly because more water molecules will bind to the adsorption sites. In order to ensure low energy consumption in practical applications, the continuous dehumidification performance of fabric during energy exchange needs to be considered. Therefore, Figure S3 (Supporting Information) examined the cycling stability of its moisture adsorption–desorption process at 60% RH. After nine cycles, the fabric retains its initial adsorption level of 0.96 g g^−1^. This means that, unlike dissolvable hygroscopic salts, Zn‐complex exhibits no mass loss with the accumulation of liquid water. Its ability to stay stably anchored on the microfiber backbone after absorbing water ensures the fabrics' moisture absorption capacity during the continuous adsorption‐evaporation process. Its long‐term stability outperforms that of other moisture‐absorbing agents previously reported in the literature. To further evaluate the advantages of the asymmetric fabric in moisture absorption, the saturated moisture absorption was quantitatively compared with most of the moisture‐absorbing materials reported in the literature (Figure 3c). It is clear that the adsorption zone behaves better at absorbing moisture compared to other moisture‐absorbing materials. As a result, the ABMTF can meet the demands of practical applications. After moisture absorption, the fabric was gradually wetted, and the color of the moisture absorption zone gradually became lighter, while the photothermal zone's black color gradually deepened (Figure S4, Supporting Information). These distinct properties indicate that the asymmetric fabric is an energy‐efficient material capable of converting gaseous water to liquid water without consuming any energy.

Considering that moisture/thermal management fabric work through a continuous adsorption–evaporation process, we investigated the photothermal evaporation performance of asymmetric fabrics. In this instance, the fabric's a‐MWCNTs‐impregnated side serves as the photothermal zone to achieve water vapor evaporation from the absorbed moisture. The uniform loading of a‐MWCNTs on microfiber surface increases the light scattering of incident light within the photothermal layer and improves the absorption of broadband solar radiation. The heat generated is effectively confined within the photothermal region with minimal heat transfer to the absorption region, enhancing water evaporation. In the standard solar spectrum (AM1.5 G), the photothermal zone exhibits high absorption (96%) in the broad wavelength range of 250–2500 nm (Figure 3d). The asymmetric fabric with strong broadband solar absorption, fast moisture transport, and good moisture permeability achieves enhanced water vapor evaporation under 1 solar illumination (Figure 3e). As shown in Figure 3f, the water molecules quickly spread toward the photothermal zone after impregnating the water supply side with blue ink. After 1 sun irradiation, the diffusion rate is accelerated and the diffusion front is quickly directed to the photothermal zone, indicating that the ADF has strong photothermal evaporation capability.

To examine the importance of the photothermal zone, we used an IR thermal camera to record the surface temperature distribution of the pure cellulose fabric (left side) and the photothermal zone (right side) under 1 solar illumination (Figure 3g). The initial surface temperatures of pure cellulose fabric (26.0 °C) and photothermal zone (26.0 °C) are almost identical before light exposure. But within a few minutes, the surface temperature of the photothermal zone rise quickly, reaching 75.0 °C after 10 minutes (Figure 3h). Under solar illumination, the ADF exhibits good local thermal properties, demonstrating that the material possess high photothermal conversion to promote solar‐driven water evaporation. To study the evaporation performance, the continuous mass loss was measured at 1 kW m^−2^ for both pure cellulose fabrics and ADF. The evaporation rate of 1.39 kg m^−2^ h^−1^ for the ADF is much higher than that of cellulose fabric (0.46 kg m^−2^ h^−1^) (Figure 3i). Thus, the above results indicate that the ADF with superior moisture permeability, good light absorption, and high photothermal conversion shows fast evaporation rate under weak solar irradiation. Figure 3j presents the working duration of the moisture management fabrics loaded with different desiccants (CaCl_2_ and Zn‐complex nanosheets). No salt accumulation behavior can be found in Zn‐complex nanosheets impregnated fabrics after 5 h of continuous evaporation under 1 sun radiation.

### 2.4 Photothermal Dehumidification Performance of Moisture/Thermal Management Fabric

**Figure 4:**
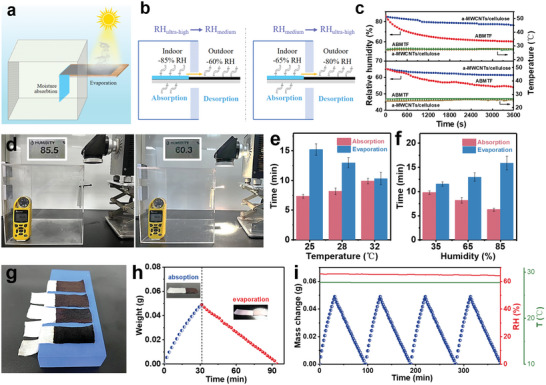
Photothermal dehumidification performance moisture/thermal management fabric. a) Schematic illustration of the solar‐driven moisture pump model. b) Diagrams showing how the moisture pump model moves moisture to achieve indoor dehumidification. c) Indoor relative humidity and temperature over time under one‐sun illumination for these devices placed in different surroundings were recorded. d) Experimental setup used to test the effectiveness of moisture pump on reducing indoor humidity. Evaporation and regeneration time for the moisture pump to produce 0.01 g mass change e) as the environmental temperatures rise from 25 °C to 32 °C and f) as the RH increase from 35% to 85%. g) Physical pictures of asymmetric dehumidification fabric. h) Mass increase of the fabric (30 × 90 mm, 30 wt% Zn‐complex) at 55% RH, followed by evaporation at 25 °C with 55% RH. i) Repeated mass change during the moisture absorption and evaporation processes.

The designed ABMTF was used as a solar‐driven dehumidification pump to evaluate its practical application. To investigate its continuous dehumidification performance, a solar‐driven dehumidification model was built. **Figure** **4**a depicts a schematic diagram of the moisture pump model driven by simulated solar illumination. When light projected by the solar simulator illuminates the ABMTF's photothermal zone, indoor moisture will be pumped by the fabric and transported toward outdoors in the form of water vapor. Figure 4b shows that moisture actively transfer from an ultrahigh‐humidity to a medium‐humidity environments and also from a medium‐humidity to an ultrahigh‐humidity environments through the ABMTF under sunlight illumination. The ABMTF has an unique advantage over conventional indoor desiccants in terms of its dehumidifying ability. To assess the dehumidification efficiency of the fabric, we evaluated the RH reduction in a confined space where the ABMTF absorbs indoor moisture and evaporates it to the outdoors (Figure 4d). When the house model was placed in a constant 70% RH outdoor environment, the ABMTF lowered the indoor RH from 85.0% to 60.0% within 1 h, while the desiccant‐free fabric reduced the indoor RH to 80.1%. When the indoor humidity was 65%, the RH could also be quickly reduced to 55% using the photothermal dehumidification fabric (Figure 4c). It is noteworthy that the ambient temperature remained nearly constant, with only a minor temperature change from adsorption heat. Overall, the ABMTF shows excellent dehumidification performance, which is significantly higher than the desiccant‐free photothermal fabric, indicating that Zn‐complex nanosheets play a crucial role in achieving fabric's photothermal dehumidification. The regulated indoor humidity using the moisture management fabric meets the human body's need for comfortable living (40–60% RH).^[^ ^49^, ^50^ ^]^

Noteworthy, the ABMTF achieves efficient and continuous indoor dehumidification under one sunlight illumination, regardless of the outdoor RH. As shown in Figure 4d, the model device for this dehumidification testing consists of a house model (glass container), a sunlight simulator, and a temperature/humidity data logger. The cubic glass container with dimension of 300 × 300 × 300 mm was sealed with sealing tape, one piece of the ABMTF (30 × 90 mm) installed on the window to simulate indoor dehumidification (the indoor moisture absorption area is 30 × 60 mm, the outdoor photothermal evaporation area is 30 × 30 mm). The temperature/humidity recorder measured an initial indoor ambient RH of 85%, which quickly dropped to 60% after starting sunlight illumination.

In order to further analyze the moisture absorption–evaporation performance of the ABMTF, the moisture absorption–evaporation rates were examined at various temperature/humidity levels. Figure 4e,f show the time required for the evaporation regeneration of the asymmetric fabric after absorbing 0.01 g of moisture under various environmental conditions. The ABMTF can continuously adsorb and release moisture under different temperature/humidity conditions, indicating its dehumidification will adapt to various outdoor environments. As can be inferred from the findings, low temperature and high humidity conditions are more conducive to moisture absorption, while high temperatures make it easier for moisture evaporation. As shown in Figure 4h, the dried ABMTF continuously absorbs moisture at 27 °C and 65% RH, increasing its mass from 0 to 0.05 g in 31 min. When the photothermal zone is irradiated by 1 sun, the fabric continues to lose moisture and the mass is returned to the original dry weight in about 63 min. By switching back and forth between moisture absorption and evaporation, the processes exhibit high cyclic stability (Figure 4i). The same moisture pump can be simply replicated to achieve excellent scalability for large‐scale indoor dehumidification (Figure 4g). Hence, the asymmetric fabric is expected to improve indoor humidity suitable for comfortable human living with zero energy consumption.

### 2.5 Transpiration‐Driven Power Generation Behavior of the Asymmetric Fabric

**Figure 5:**
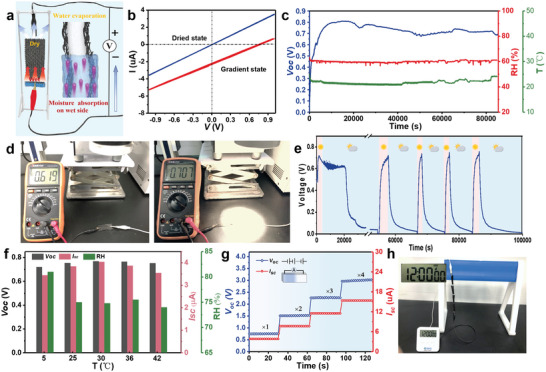
Transpiration‐driven power generation behavior of the asymmetric fabric. a) Schematic illustration of the working principle of transpiration‐driven TEPG constructed in evaporation zone. b) *I*–*V* curves of the dried evaporation zone (blue line) and the evaporation zone with gradient water (red line). c) Continuous *V*_oc_ measurement under constant ambient temperature and humidity. d) Measured *V*_oc_ changes of TEPG when illuminating the drying sides of evaporation zone with 1 sun intensity. e) Electric output of the TEPG in response to light irradiation. The TEPG is electrically regenerated after sunlight irradiation. f) The TEPG work across a wide temperature and humidity range. g) *V*_oc_ and *I*_sc_ were scaled up by connecting multiple TEPG in series and in parallel. h) Direct electrical output is demonstrated by connecting four TEPG to illuminate a miniature electronic watch.

Based on the excellent performance of asymmetric moisture management fabric, we expect a broad application of such fabric and propose another application model. As shown in **Figure** **5**a, a transpiration‐driven electrokinetic power generator (TEPG) that uses the absorbed moisture in absorption zone to generate electricity was successfully constructed. After water absorption in the desiccant loading zone, it diffuses into the a‐MWCNTs loaded evaporation zone, generating a continuous voltage and current between the wetting and drying sides of the evaporation zone. As hydrophilic fabrics facilitate fast capillary flow, when water flows from the wetting side to the drying side, the a‐MWCNTs loaded wet region produce fixed −COO− and −O− groups and free protons (H^+^) on carboxyl and hydroxyl groups of a‐MWCNTs and cellulose fabrics, respectively.^[^ ^51^, ^52^ ^]^ Protons and solvated cations on the wetting side and electrons on the drying side of the carbon/water interface are transported through the capillary flow of water. The electrons in the wet carbon are transported in the water flow direction, which means that a pseudocurrent is generated. Due to the simultaneous moisture absorption–evaporation process of the asymmetric fabric, a natural water gradient forms between the wetting and drying sides, and protons are constantly migrating from the wetting side to the drying side. The drying side creates the positive terminal, whereas the wetting side creates the negative terminal. As a result, the TEPG system can continuously generate current and voltage.

According to our results, during water absorption, the fabric gave rise to the development of the open‐circuit voltage (*V*_oc_), yielding a *V*_oc_ of ≈0.78 V after 43 min of moisture absorption (Figure S5, Supporting Information). Figure 5b provides a systematic overview of the electrical behavior of the fabric generator. First, the a‐MWCNTs‐loaded evaporation zone with a resistance of 52 MΩ exhibits typical ohmic behavior in drying state. In contrast, after wetting the left evaporation zone, the fabric exhibits typical energy generator characteristics, generating an *V*_oc_ of ≈0.78 V and a short‐circuit current (*I*_sc_) of ≈2.22 µA. Unlike most previous moisture‐driven electric generators, which require specific environmental conditions to operate and can only provide transient electrical response, TEPG is able to maintain their continuous power performance due to their permanent wet‐dry asymmetry. As shown in Figure 5c, the generator's *V*_oc_ is consistently maintained in the 0.7–0.8 V range for up to 24 h at 23 °C and 60% RH. The results in Figures S6 and S7 (Supporting Information) demonstrate that only when the evaporation zone forms a water gradient between the dry and wet states can the fabric perform as a fully running generator. In fact, the amount of water absorbed in the absorption zone influences the water gradient formation and determines the fabric's electrical generation. The *V*_oc_, *I*_sc_, and electric power generated (P) of TEPG depend on its internal resistance (Figure S6b, Supporting Information), which can be controlled by a‐MWCNTs loading. The evaporation‐driven continuous capillary flow generates a *V*_max_ of 0.82 V, and a *P*_max_ up to 1.13 µW cm^−3^. When continuous light is introduced in the evaporation zone, water gradient can be promoted and is expected to be restored again even when the fabric is completely wetted by water. To confirm this, the evaporation zone surface was irradiated by a solar simulator with 1 sun intensity, and the *V*_oc_ changes of the generator were tracked. As shown in Figure 5d, when the photothermal zone with a resistance of 42 MΩ forms a stable water gradient, its *V*_oc_ measured by multimeter can be up to 0.62 V. When illuminating the drying end, its *V*_oc_ rapidly increases to 0.71 V, indicating that the introduction of light can enlarge the water gradient and increase the power generation. After removing the light, the *V*_oc_ gradually recover to 0.62 V. Following a period of moisture absorption, the water gradient in the evaporation zone drop to a lower level and the *V*_oc_ further decreases to 0 V. However, the voltage quickly return to ≈0.71 V after applying one sun illumination once more, and it will return to 0 V after turning off the light (Figure 5e). By switching the light on and off in a continuous cycle, the *V*_oc_ of the fabric fluctuate frequently, indicating that the fabric has good hydrolysis and structural stability, as well as good reusability. It is worth noting that the generator can still operate over a wide temperature range if RH is kept within a suitable range (Figure 5f). This is because the fabric could continue to generate power as long as it continue to absorb moisture and create a water gradient. The expanded output of electrical energy from fabrics can be achieved by connecting them in series and parallel. Specially, series connections of fabrics can increase *V*_oc_, while parallel connections (stacking) can increase *I*_sc_. Such connections can simplify the application of fabric generators for scale‐up (Figure 5g). The assembled fabric generators can be used directly for energy supply. After absorbing water in the photothermal zone to create a water gradient, four freshly created fabric generators can be connected in series to power miniature electronic watch (Figure 5h). As a result, the asymmetric fabric can use abundant solar energy resources and natural water evaporation to achieve electricity generation, opening up a new avenue for the high‐efficiency utilization of environmental energy.

### 2.6 Zero‐Energy Radiant Cooling Performance of Moisture/Thermal Management Fabric

**Figure 6:**
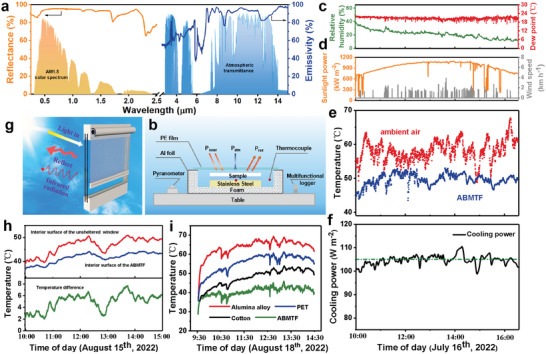
The optical properties and passive radiative cooling performance of the moisture/thermal management fabric. a) Spectral UV–visible–infrared reflectance/emittance of the ABMTF's radiant layer. b–e) Real‐time temperature measurement for the ABMTF, along with the outdoor meteorological conditions in Chongqing (date, July 16, 2022, UTC+8). b) Schematic of the setup for testing the radiative cooling temperature. c) Relative humidity (RH; green curve, left axis) and dew point (red curve, right axis). d) Solar irradiance intensity (yellow curve, left axis) and wind speed (gray curve, right axis). e) Real‐time temperature changes between10:00 a.m–16:30 p.m. in this clear‐sky day for the ABMTF and ambient environment. f) Profile of the measured cooling power. g) Illustration of the ABMTF's operation mechanism as a cooling curtain. h) Comparison and calculated temperature difference of the window interior temperatures between the sheltered and unsheltered scale‐model buildings. i) The temperature evolution of the window interior with different sheltering ways under sunlight.

To endow moisture management fabric with passive cooling function, we designed an asymmetric bilayer structure where the functions of thermal radiation reflection, and mid‐IR emission were coupled to the fabric to manipulate unbalanced heat transmission. Such multimodal textile was constructed by spraying a radiative CA layer on one side of the ADF. The inherent C−O, C−O−C, OH, C=O, and CH_2_ multiple bonds of CA molecules endow them broadband and high mid‐IR emissivity, and the water‐assisted induced phase separation after rapid spraying customizes the porosity for scattering incident solar radiation to achieve high‐performance large‐scale cooling.^[^ ^28^, ^53^ ^]^ The optical properties of the CA radiative layer were tested using ultraviolet–visible‐near infrared (UV‐Vis‐NIR) and FTIR spectra equipped with integrating spheres. **Figure** **6**a shows that the CA layer displays a high reflectance of over 96.3% in the solar light region (0.2–2.5 µm) and a high mid‐IR emissivity of 92.1% in the 7–15 µm range, making it an ideal material for radiative cooling. A porous CA layer with a certain thickness can suppress the light absorption through multiple Mie scattering. We further sprayed 53, 83, 121, and 219 µm thick CA radiative layers on fabric, respectively, and measured their corresponding solar spectral reflectance (Figure S8, Supporting Information). The reflectivity of ABMTF is positively correlated with the CA layer thickness, reaching 95% at 83 µm thickness, and then changes weakly above 83 µm thickness. The high mid‐IR emissivity of CA layer is closely related to its penetrating porous structure from the surface to the interior, which can provide excellent impedance matching with air, effectively reducing the IR reflection, i.e., the opening porous structure can increase the relatively high transmissivity of bulk CA.

To experimentally verify the radiative cooling performance of the ABMTF, we conducted real‐time outdoor measurements of temperature and cooling power during a sunny day of July 16, 2022 in Chongqing, China. As shown in Figure 6b, the device consists of an acrylic housing covered with a layer of aluminum foil, a foam‐insulated sample stage, a stainless steel plate, and an IR‐transparent polyethylene (PE) film. In Figure 6c–e, we recorded the ABMTF's temperature changes as well as the daytime outdoor meteorological conditions. As a reference, Figure 6c shows the outdoor RH and dew point for different time periods. Figure 6d shows the solar power and wind speed curves. The real‐time temperature tracking of the environment and ABMTF are shown in Figure 6e. From 10:00 am to 16:30 pm, it is obvious that the ABMTF is consistently cooler compared to the ambient temperature. During the testing period, the sky was clear, the average solar irradiance was 800 W m^−2^, the wind speed was 1.2 km h^−1^, and the average RH was 25% (Figure 6d). The subambient temperature of the ABMTF drop by |Δ*T*| ≈12 °C even at midday solar intensity peaks close to 1000 W m^−2^ (Figure 6e). The corresponding cooling power of the ABMTF measured by thermal compensation method was up to 106 W m^−2^ (Figure 6f). The results confirm the excellent cooling capacity of the customized asymmetric bilayer fabric, which prevents heating from solar irradiance while effectively releases heat  through IR radiation.

As proof of concept, we demonstrated its application as cooling curtain by covering ABMTF (with the radiant layer facing outdoors) on the window interior for reducing heat input from the outdoors (Figure 6g). As shown in Figure 6h, the interior surface temperature of installed ABMTF is consistently below the unsheltered window interior during the testing period, with a subambient temperature reduction ranging from 3 °C to 7 °C. We monitored the temperature evolutions of window interior sheltered by different commercial curtain materials under 5 h of sunlight illumination (from 9: 30 am to 2: 30 pm on August 18, 2022 in Chongqing, China). Compared to commercial aluminum, polyester, and cotton fabric‐based window coverings, the ABMTF exhibits superior cooling effects. The temperature of the ABMTF is 42 °C, compared with up to 67 °C, 59 °C, and 50 °C for aluminum, polyester, and cotton fabrics at 12:00 am, respectively (Figure 6i). The passive temperature reduction not only saves a significant amount of energy for cooling but also helps to achieve the Net Zero Carbon 2050 goal.

## 3 Conclusion

In summary, we report a zero‐energy multimode moisture/thermal management fabric with an asymmetric bilayer design using a facile, scalable two‐step dip‐coating and spraying method. It provides triple function: (1) continuous indoor dehumidification under solar irradiation, (2) power generation using the transpiration‐driven electrokinetic effect, and (3) passive radiative cooling by reflecting sunlight and emitting mid‐IR light using the spectrally selective CA layer. Zn‐complex nanosheets are firmly adhered to the multiscale micro‐nano‐fiber backbone in absorption zone for super‐hygroscopicity and excellent cyclability. The hydrophilic evaporation zone forms a highly connected dual network of 3D a‐MWCNTs and cellulose microfibrils, exhibiting 96% high broadband solar light absorption and good moisture permeability, ensuring efficient photothermal conversion and promoting moisture evaporation. The CA radiant layer exhibits a high reflectance in the sunlight region due to disordered network structure, and a selective emittance in the mid‐IR region in light of its specific bond vibrations.

The experimental results showed that the asymmetric fabric based moisture pump was able to quickly reduce the high indoor RH to a moderate level under 1 sun illumination to meet the human comfort needs. The transpiration‐driven ion flow during moisture pump operation promotes electro‐kinetic power generation. A single self‐powered fabric is found to produce a maximum *V*_oc_ of ≈0.83 V, a maximum *I*_sc_ of ≈5.51 µA, and a maximum power density up to 1.13 µW cm^−3^. Finally, four fabrics connected in series capable of powering a miniature electronic timer with zero energy input. Real‐time temperature measurements show that the zero‐energy designed cooling fabric exhibits high passive cooling capability under 1 solar radiation with a midday absolute temperature difference of ≈12 °C. In addition, the results show that covering the window with ABMTF prevent overheating compared with traditional curtains. To the best of our knowledge, this is the first reported multimode fabric that combines indoor dehumidification, transpiration‐driven power production and radiation cooling functions. The facile fabrication process, combined with our fabric's low cost (Tables S1 and S2, Supporting Information), positions our ABMTF fabric as a competitive candidate for energy‐efficient air‐conditioning applications. We envision such a zero‐energy multimode fabric would have great and practical potential for global thermal and humidity management as well as energy saving applications, and offers a renewable zero‐energy platform for achieving the goal of Net Zero Carbon 2050.

## Conflict of Interest

The authors declare no conflict of interest.

## Supporting information

Supporting Information

## Data Availability

The data that support the findings of this study are available from the corresponding author upon reasonable request.
