# Supplementary material for: A Zero‐Energy, Zero‐Emission Air Conditioning Fabric
Source: Adv Sci (Weinh). 2023 Feb 15;10(11):2206925. doi: 10.1002/advs.202206925 (PMC10104666; doi:10.1002/advs.202206925)
Supplement: Supplementary file 1 — Supporting Information [file ADVS-10-2206925-s001.pdf]

Supporting Information

Title: **A zero-energy, zero-emission air conditioning fabric**

Kai Zhang,[1,*] Xiaojuan Lei,[2] Caiqing Mo,[1] Jin Huang,[3] Ming Wang,[3] En-Tang Kang,[1] Liqun Xu[1,*]

Affiliations

[1]School of Materials and Energy, Chongqing Key Laboratory of Soft-Matter Material Chemistry and Function Manufacturing, Southwest University, Chongqing 400715, P. R. China

[2]College of Food Science, Chongqing Key Laboratory of Soft-Matter Material Chemistry and Function Manufacturing, Southwest University, Chongqing 400715, P. R. China

[3]School of Chemistry and Chemical Engineering, Chongqing Key Laboratory of Soft-Matter Material Chemistry and Function Manufacturing, Southwest University, Chongqing 400715, P. R. China

Corresponding author: kaizhang095@swu.edu.cn; xulq@swu.edu.cn

Supporting Note

1. Experimental Methods:

1.1 Manufacture of dehumidified Zn-complex nanosheets. Zn-complex nanosheets were synthesized by a time-saving and economical method. Briefly, Zinc-complex are obtained by the coordination reaction between ethanolamine ligands and zinc chloride salts. 0.08 M of anhydrous zinc chloride was dissolved in 100 mL of ethanol. Under stirring, 0.04 M ethanolamine was added dropwise to the $ZnCl_2$ solution. Then the Zn-complex was obtained by the unsaturated coordination of ethanolamine ligands with zinc ions under continuous stirring for 30 min. Finally, the mixture was poured into a surface dish and dried overnight at 90°C under vacuum to obtain the milky white Zn-complex nanosheets.

1.2 Preparation of asymmetric Zn-complex/a-MWCNTs/cellulose layer (ADF). The a-MWCNTs and Zn-complex nanosheets were coated onto the cellulose fabric skeleton by dipping method. Firstly, a-MWCNTs and Zn-complex were added into deionized water, respectively, and then dispersed under ultrasonic treatment for 1 h to obtain stable 3 wt% aqueous a-MWCNTs dispersion and 5 wt% Zn-complex dispersion. Subsequently, the

unilateral side of the hydrophilic cellulose fabric was dipped into the prepared a-MWCNTs dispersion, impregnated for 10 min and then dried. After several soaking-drying cycles, the a-MWCNTs coated cellulose fabrics were obtained. Finally, the Zn-complex nanosheets were effectively deposited on the other side of the cellulose fabric in the same way to prepare the asymmetric structured cellulose/Zn-complex/a-MWCNTs moisture management layer.

1.3 Preparation of asymmetric bi-layer cellulose/Zn-complex/a-MWCNTs/CA fabric. 15 wt% of CA was added into 100 mL acetone and was vigorously stirred at room temperature for 6 h. Then the obtained CA solution was quickly sprayed onto the surface of Zn-complex/a-MWCNTs/cellulose fabric by using an electronic spray gun for 5 min. The ambient temperature and RH were maintained at 25 ± 1 °C and 52 ± 3%, respectively. The sprayed fabric was then submerged in deionized water for 6 min. Finally, the prepared asymmetric bi-layer cellulose/Zn-complex/a-MWCNTs/CA fabric (ABMTF) were dried at 100 °C under vacuum.

2. Characterization

The microstructures of the Zn-complex nanosheets, a-MWCNTs, pure cellulose fabrics, ADF and ABMTF were characterized by FE-SEM (JSM-7800F) equipped with energy disperse X-ray spectroscopy. FT-IR spectra of the complex and fabrics were measured by an FT-IR spectrophotometer (Agilent CARY 660). The reflectance and transmittance spectra were measured in the UV-Vis-NIR range from 250 to 2500 nm using a UV/Vis spectrometer equipped with an integrating sphere (Hitachi, UH4150, Japan). Mid-infrared spectral reflectance and transmittance at 2500-15000 nm were obtained by an FTIR spectrometer equipped with a gold integrating sphere (Nicolet 6700, Thermo Fisher Scientific, USA). Raman spectra were recorded using a Raman spectrometer (inVia-Reflex) with an excitation laser of 532 nm. The contact angle of the fabric was measured using droplet shape analysis (OCA25, Dataphysics).Thermal images and surface temperature distributions of the fabrics were recorded by an infrared thermal imaging camera (TiS75, Fluke, USA). The relative humidity and temperature of the environment were monitored using a thermohygrometer (CENTER 310). Material temperature was measured using a thermocouple (TT-K-30, Omega Corporation) and recorded by a data logger (TC-08, Pico Technology). Prior to testing, all samples were dried under vacuum at 100 °C for 5 h.

3. Performance Testing

3.1 Moisture Absorption/Evaporation Measurement. Both moisture absorption capacity and cycling stability tests were studied using a constant temperature and humidity chamber. Moisture absorption tests were conducted at 25 °C and at various humidity levels (60, 70, 80 and 90% RH). Before testing, the absorption zone with dimension of 35 mm×40 mm were cut and dried under vacuum at 100 °C until their weight remained constant and then placed in a chamber with constant temperature and humidity. The moisture absorption process of the fabric was real-time recorded by weighing on a communicable analytical balance (METTLER TOLEDO 104E). During the moisture absorption-desorption cyclic tests, the material was kept at 25 °C and 70% RH for 6 hours to absorb moisture, and then dried at 100 °C for 1.5 hours to release moisture. The moisture absorption capacity of the samples was calculated according to the following equation:

$$C_{abs} = \Delta m / \mathrm{m}_0$$

Where C_{abs} denotes the moisture absorption capacity based on unit weight of cellulose/Zn-complex absorption zone (g g^{-1}), Δm implies the moisture absorption quantity (g), and m_0 indicates the mass of the dried absorption zone (g).

Solar evaporation experiments were conducted in a chamber at constant temperature (~25°C) and RH (~50%), using a standard solar simulator (CEL-S500) with a controlled solar irradiance of 1 kW m^{-2}. The constant light intensity was imposed by simultaneously adjusting the current of the simulator and the distance between the fabric surface and the light source, and was calibrated by utilizing a solar power density meter (JTTS-01). Prior to solar evaporation measurements, the samples were saturated with moisture at 25 °C and 70% RH. The real-time mass change of the sample was recorded by an analytical balance, which was used to evaluate the water evaporation rate (kg m^{-2} h^{-1}). Sunlight-driven indoor dehumidification simulations were conducted in a constant temperature and humidity chamber. Under 1 kW m^{-2} light irradiance, the real-time RH and temperature variations of the chamber environment were monitored using a temperature and humidity meter (CENTER 310) and a Kestrel 5000 Environmental Meter.

3.2 Solar-driven Electrokinetic Power Generator. The a-MWCNTs loading in photothermal zone is controlled by varying soaking times to achieve resistance change to create the TEPGs with desired V_{OC}. Cyclic voltammetry (CV) curve, V_{OC} and short-circuit current (I_{SC}) of the fabric's photothermal region were measured using a digital source meter

(2400, Keithley). All experiments were performed at 50% relative humidity (RH), except for humidity control measurements.

3.3 Passive Radiative Cooling Measurement. The cooling temperature of the asymmetric double-layered fabric was measured by the device shown in Figure 6b. During the experiments, parasitic conduction and convection were minimized to reduce the heat losses. Our thermal box was made up of insulating foam that was wrapped with reflective Al foil. A 10-um-thick transparent low-density polyethylene wind cover was utilized to seal the thermal box. A 50 mm×50 mm fabric was placed on a 50 mm×50mm×1mm stainless steel plate. The device was placed on a 1.2 m high table to prevent heat conduction from the ground to the thermal box.

To detect the real-time temperature of the sample, a K-type thermocouple was directly installed on the back side of the textiles and was continuously recorded by a datalogging thermometer. For comparison, a thermocouple was mounted in the indoor locations to detect real-time temperature of the ambient. The cooling power is measured by a device with a temperature feedback circuit. The temperature of the fabric is controlled to be equal to the ambient temperature by means of a silicone heating pad, and the heating power of the fabric is used to compensate for the cooling power of the fabric, so that the cooling power is equal to the heating power. This method is consistent with the results of previous studies. The heating power of the fabric was monitored and recorded by a power meter (66205, Chroma). Incident solar power, wind speed, dew point, relative humidity and ambient temperature during the measurements were also recorded.

Supporting Figure S1-S8 and Table S1-S2

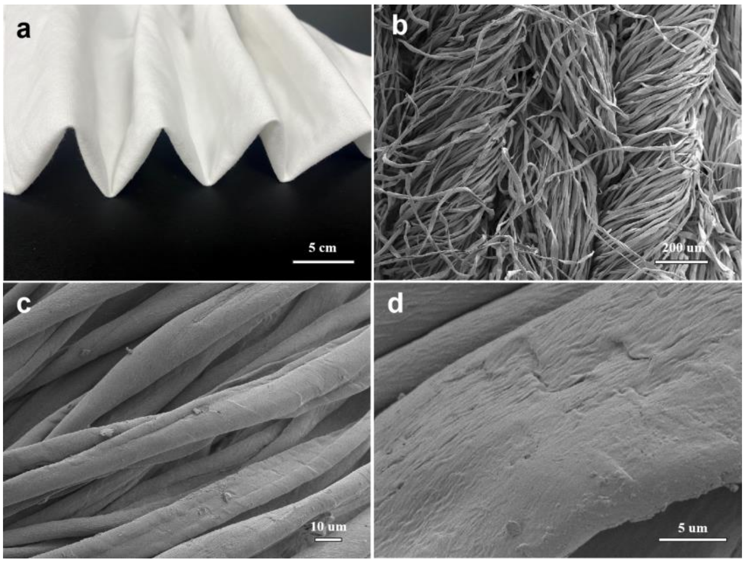

Figure S1. a Photographs of flexible and large-scale cellulose fabric. **b-c** FE-SEM images of cellulose fabric composed of entangled cellulose microfiber bundles. **d** FE-SEM image of partially aligned nanofibers in cellulose microfibers.

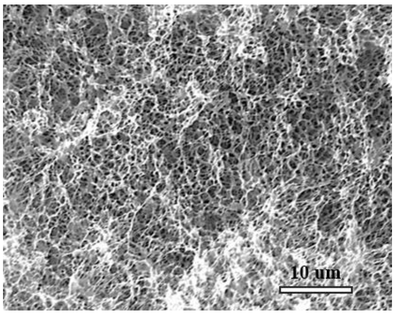

Figure S2. The cross-sectional SEM images of the radiative layer.

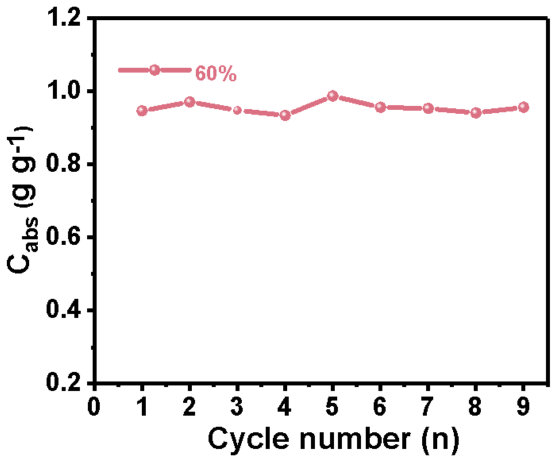

Figure S3. Cycling stability of moisture absorption-desorption of absorption zone

(cellulose/Zn-complex) at 25 °C and 60% RH (desorption at 100 °C).

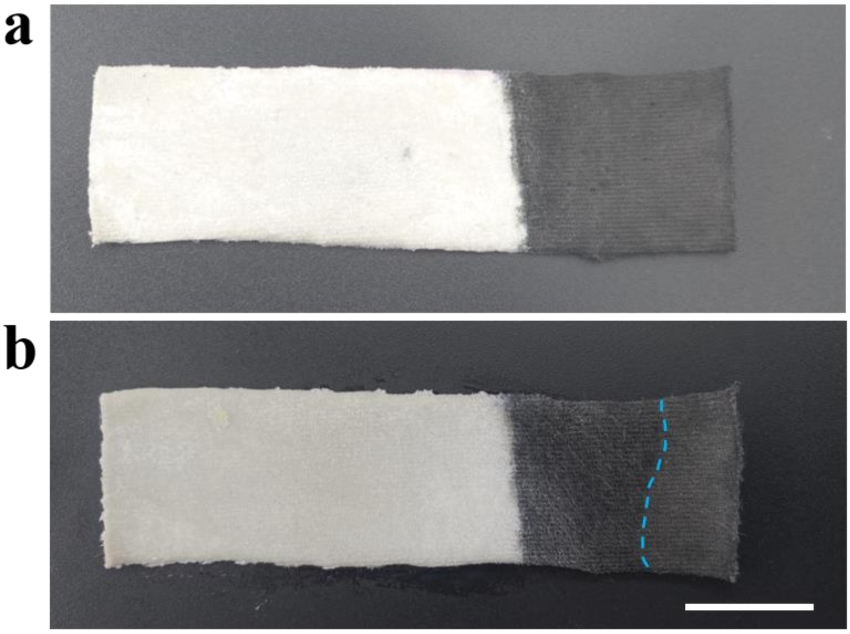

Figure S4. Optical images showing the moisture absorption, water wetting, and color change of ADF at 25 °C and 80% RH. The scale bar is 2 cm.

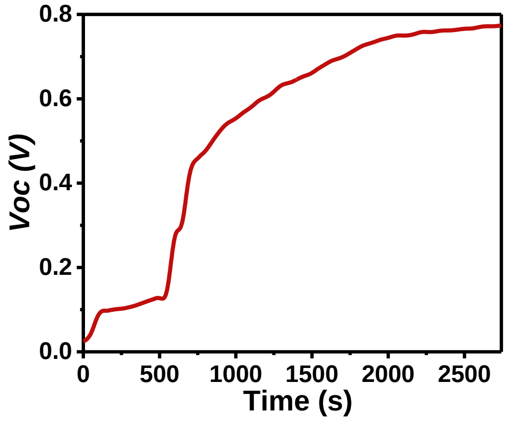

Figure S5. The open-circuit voltage (V_{oc}) change of the asymmetric fabric during the whole initial water absorption process at 25 °C and 75% RH.

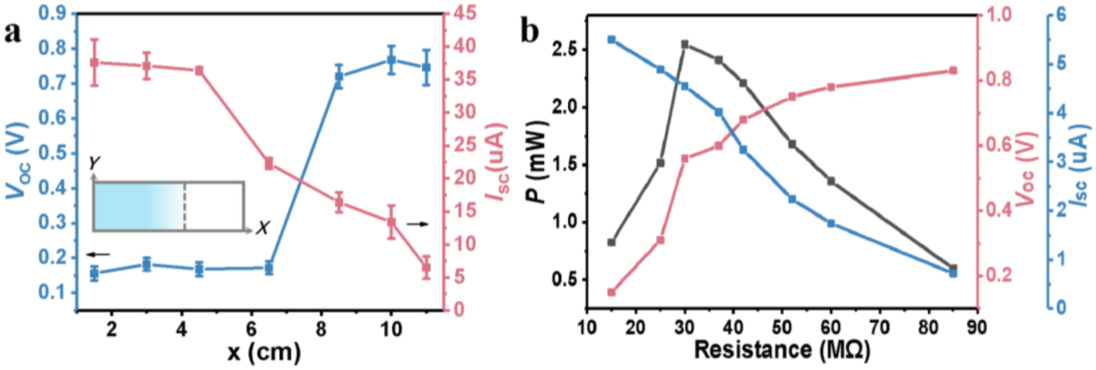

Figure S6. a The correlation between output voltage (blue curve), current (red curve) and the measuring distance between wet-carbon and dry-carbon of the TEPG. **b** Measured P, I_{SC}, and V_{OC} of TEPG with different resistances.

As shown in Figure S6a, the electrical output of the photothermal evaporation zone requires that one electrode must be connected to the wet zone and the other must be connected to the dry zone. It is noteworthy that connections within the same area do not lead to any response, indicating that the water-containing gradient is crucial for electrical output. It is also observed that the generated voltage is independent of the measured distance. However, I_{SC} decreases with increasing distance. The evaporation zone's internal resistance, which is regulated by the conductive a-MWCNTs's loading mass, has a distinctive impact on power production. The P, I_{SC}, and V_{OC} of TEPG were examined at different resistance range by controlling the a-MWCNTs loadings (Figure S6b).

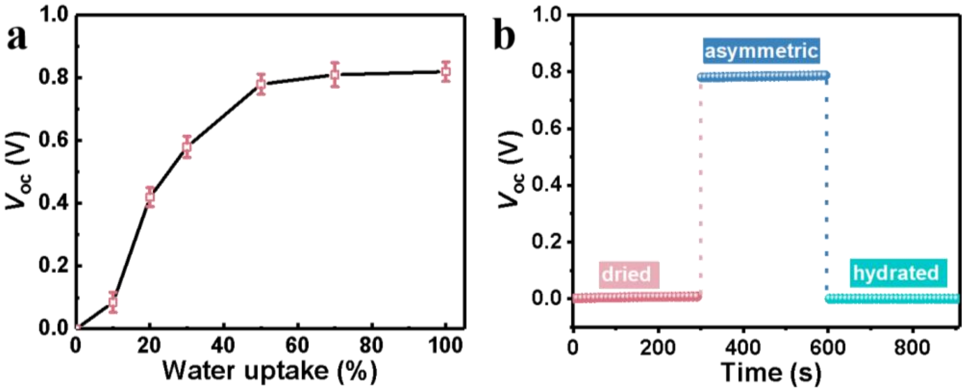

Figure S7. a V_{oc} as a function of the water uptake amount of the asymmetric fabric. **b** Electric performance of TEPGs in response to varying water gradient state.

In addition, when the fabric is exposed to moist air, its electricity production rises gradually with the increase of water intake. Once the water intake in the moisture-absorbing zone reaches a certain level, and a sufficient water gradient is formed on both dry and wet sides, its voltage no longer changes with the water content (Figure S7a). Figure S7b illustrates that the photothermal zone produces zero energy when it is completely dry, and the voltage peaks when water is absorbed to form a water gradient. However, when the absorbed water completely infiltrates the evaporation zone, its voltage rapidly drops to 0 V.

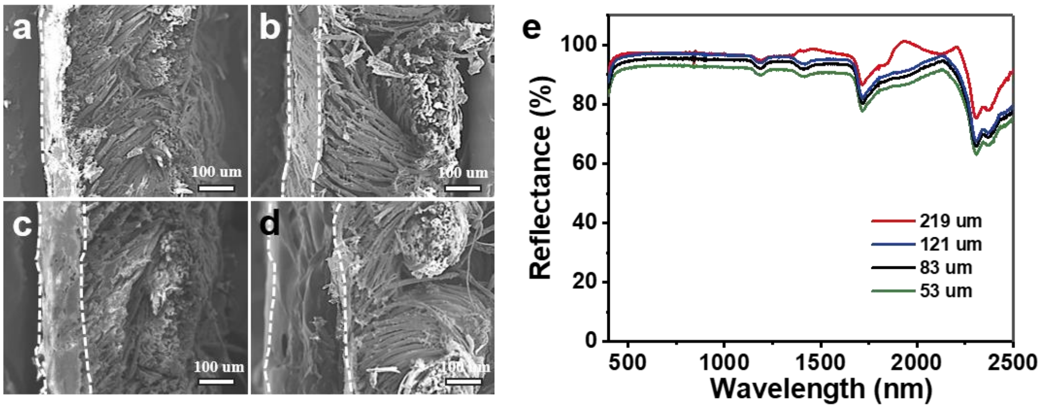

Figure S8. a-d FE-SEM images of ABMTF fabric with different thickness of CA radiation layer. **e** Spectral reflectance of the ABMTF's radiant layer.

Table S1. Input and output of the preparation of our ABMTF presented in this work.

**Step 1: The fabrication process of Zn-complex**	The preparation of Zn-complex nanosheets containing 0.016 M $ZnCl_2$, 0.008 M ethanolamine, and 20 mL ethanol. The mixture was obtained under 30 min stirring and dried overnight at 90 °C under vacuum.		
Item	Number	Units	Additional data and comments
**Input**			
$ZnCl_2$	1.090	g	
Ethanolamine	0.244	g	
Ethanol	15.786	g	
Energy[1]	0.000036	kWh	The electricity is used for stirring[1]
Energy[2]	0.000656	kWh	The electricity is used for drying[2]
**Output**			
Zn-complex nanosheets	1.334	g	
**Step2: The preparation of Zn-complex and a-MWCNTs dispersions**	The a-MWCNTs and Zn-complex were added into deionized water, respectively, and then dispersed under ultrasonic treatment for 1 h to obtain stable 3 wt% aqueous a-MWCNTs dispersion and 5 wt% Zn-complex dispersion.		
Item	Number	Units	Additional data and comments
**Input**			
a-MWCNTs	0.618	g	
Zn-complex	1.053	g	
Distill water	40	g	
Energy	0.0002	kWh	The electricity is used for dispersing[3]

**Output**			
Zn-complex dispersion	21.053	g	
a-MWCNTs dispersion	20.618	g	
**Step 3: The preparation of asymmetric ADF.**	Two sides of the length direction of the hydrophilic cellulose fabric were dipped into the prepared a-MWCNTs and Zn-complex dispersions, respectively, impregnated for 10 min and then dried. Through three soaking-drying cycles, the asymmetric ADF was obtained.		
Item	Number	Units	Additional data and comments.
**Input**			
Zn-complex dispersion	21.053	g	
a-MWCNTs dispersion	20.618	g	
Cellulose textile	0.6619	g	3 cm*9 cm
Energy	0.010584	kWh	The energy is used for drying[4]
**Output**			
Asymmetric ADF	2.3329	g	
**Step 4: The fabrication of asymmetric bi-layer ABMTF**	15 wt% of CA was vigorously stirred in acetone at ambient temperature for 40 min to dissolve. Then the obtained CA solution was quickly sprayed onto the surface of the ADF fabric. The ABMTF was finally obtained by drying at 100 °C.		
Item	Number	Units	Additional data and comments.
**Input**			
CA	0.3529	g	
Acetone	2	g	
Asymmetric ADF	2.3329	g	3 cm*9 cm
Distill water	5	g	
Energy1	0.000006	kWh	The energy is used for stirring[5]
Energy2	0.000440	kWh	The energy is used for drying[6]
**Output**			
Asymmetric bi-layer ABMTF	2.6858	g	3 cm*9 cm

[1]The power of the agitator is 72 W, the maximum stirring capacity is 20 L, and the stirring time is 30 min. Thus, the energy consumption of the stirring process is 30/60*0.072*0.02/20 = 0.000036 kWh.

[2]Drying stage (the specific heat of ethanol is 2400 J/Kg °C). (1) Heating stage: The electricity consumption for heating 20 mL ethanol is 65*2400*0.015/3600000 = 0.00065 kWh. (2) Heat preservation stage: 20 mL of 90 °C ethanol solutions in the laboratory at 25 °C at room temperature drops to about 0.6 °C/min. Heat loss: 2400*0.015*0.6 = 21.6 J; The continuous heating time of the heater per minute is 21.6/800 = 0.027 s. Therefore, the energy consumption in the insulation phase is 0.027/3600*0.8 = 0.000006 kWh. The energy consumption of the heater in 1 h is 0.00065 + 0.000006=0.000656 kWh.

[3]The power of the ultrasonic disperser is 100 W, the maximum stirring capacity is 20 L, and the stirring time is 1 h. Thus, the energy consumption of the dispersing process for a-MWCNTs and Zn-complex nanosheets is 2*1*0.1*0.02/20 = 0.0002 kWh.

[4]Drying stage (the specific heat of water is 4200 J/Kg °C). (1) Heating stage: The electricity consumption for heating 20 mL water is 75*4200*0.02/3600000 = 0.00175 kWh. (2) Heat preservation stage: 20 mL of 100 °C water solutions in the laboratory at 25 °C at room temperature drops to about 0.6 °C/min. Heat loss: 4200*0.02*0.6 = 50.4 J; The continuous heating time of the heater per minute is 50.4/800 = 0.063 s. Therefore, the energy consumption in the insulation phase is 0.063/3600*0.8 = 0.000014 kWh. The energy consumption of the heater in 1 h is 3*2*(0.00175 + 0.000014)=0.010584 kWh.

[5]The power of the agitator is 72 W, the maximum stirring capacity is 20 L, and the stirring time is 40 min. Thus, the energy consumption of the stirring process is 40/60*0.072*0.0025/20 = 0.000006 kWh.

[6]Drying stage. (1) Heating stage: The electricity consumption for heating 5 mL water is 75*4200*0.005/3600000 = 0.000437 kWh. (2) Heat preservation stage: 5 mL of 100 °C water solutions in the laboratory at 25 °C at room temperature drops to about 0.6 °C/min. Heat loss: 4200*0.005*0.6 = 12.6 J; The continuous heating time of the heater per minute is 12.6/800 = 0.01575 s. Therefore, the energy consumption in the insulation phase is 0.01575/3600*0.8 = 0.0000035 kWh. The energy consumption of the heater in 1 h is 0.000437 + 0.0000035) = 0.000440 kWh.

Table S2. The chemical and energy consumption cost per m^2 (or kilogram) of ABMTF fabric production.

**Parameters**	**Cost (￥)**
Cellulose textile (￥23265.3-40816.3/t)	0.015399-0.027016
$ZnCl_2$ (￥8000/t)	0.00872
Ethanolamine (￥7800/t)	0.00190
Ethanol (￥4800/t)	0.07577
a-MWCNTs (￥2500000/t)	1.54500
Water (￥1.8/t)	0.000081
CA (￥80000/t)	0.02823
Acetone (￥5950/t)	0.00960
Electric power (￥0.5489/kWh)	0.0065439
Energy cost (￥/kg)	2.4365
Chemical cost (￥/kg)	625.40-629.72
Total production cost (￥/t)	627836-632162
Total production cost (￥/m^2)	6.2453-6.2884
